# Biomarkers on Fibrotic Lung Diseases Associated with Usual Interstitial Pneumonia

**DOI:** 10.7150/ijms.129394

**Published:** 2026-04-16

**Authors:** Yuhang Cheng, Qing Chang, Feng Li

**Affiliations:** 1 Department of Basic Medical Sciences, Shanghai Jiao Tong University School of Medicine, No1, Banxia Road, Pudong new area, Shanghai, 201318, P.R. China.; 2 Department of Pulmonary and Critical Care Medicine, Shanghai Chest Hospital, Shanghai Jiao Tong University School of Medicine, No.241, West Huaihai Road, Xuhui, Shanghai, 200030, P.R. China.

**Keywords:** usual interstitial pneumonia, diagnosis, biomarkers

## Abstract

Usual interstitial pneumonia (UIP) is a common pattern in fibrotic lung diseases characterized by distinctive radiological and histopathological features. UIP is associated with various underlying causes including idiopathic UIP, i.e. idiopathic pulmonary fibrosis (IPF), and secondary UIP, e.g. connective tissue disease-associated interstitial lung diseases (CTD-ILDs) and fibrotic hypersensitivity pneumonia (fHP). Previous work suggests radiological UIP patterns have strong correlations with overall poor prognosis. This review synthesizes current knowledge on the diverse entities of fibrotic lung diseases presenting with the UIP pattern. This review also highlights previous studies in biomarker searching of IPF and secondary UIP. A number of biomarkers associated with disease diagnosis are summarized, providing new insights for clinicians in disease differentiation. Finally, this review emphasizes the future directions of differential diagnosis through radiology- and blood- biomarkers with the integration of artificial intelligence (AI).

## Introduction

**Figure 1:**
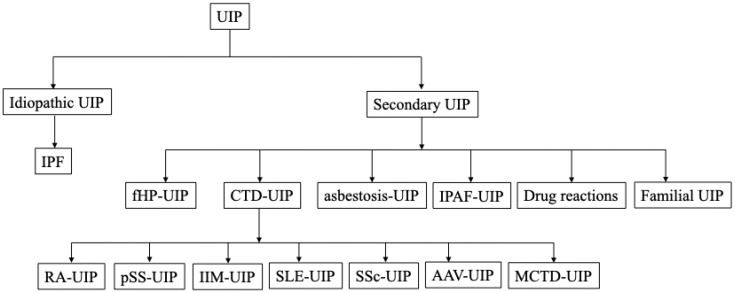
** Underlying diseases associated with a radiologic UIP pattern.** The tree diagram shows the spectrum of clinical entities that may manifest as a UIP pattern. A*bbreviations:* UIP, Usual interstitial pneumonia; IPF, Idiopathic pulmonary fibrosis; fHP, fibrotic hypersensitivity pneumonitis; CTD-UIP, Connective tissue disease-related UIP; IPAF-UIP, Interstitial pneumonia with autoimmune features related UIP; RA, Rheumatoid arthritis; pSS, primary sjögren's syndrome; IIM, Idiopathic inflammatory myopathy; SLE, Systemic lupus erythematosus; SSc, Systemic sclerosis; AAV, Anti-neutrophil cytoplasmic antibody associated vasculitis; MCTD, Mixed connective tissue disease; UCTD, Undifferentiated connective tissue disease

**Figure 2:**
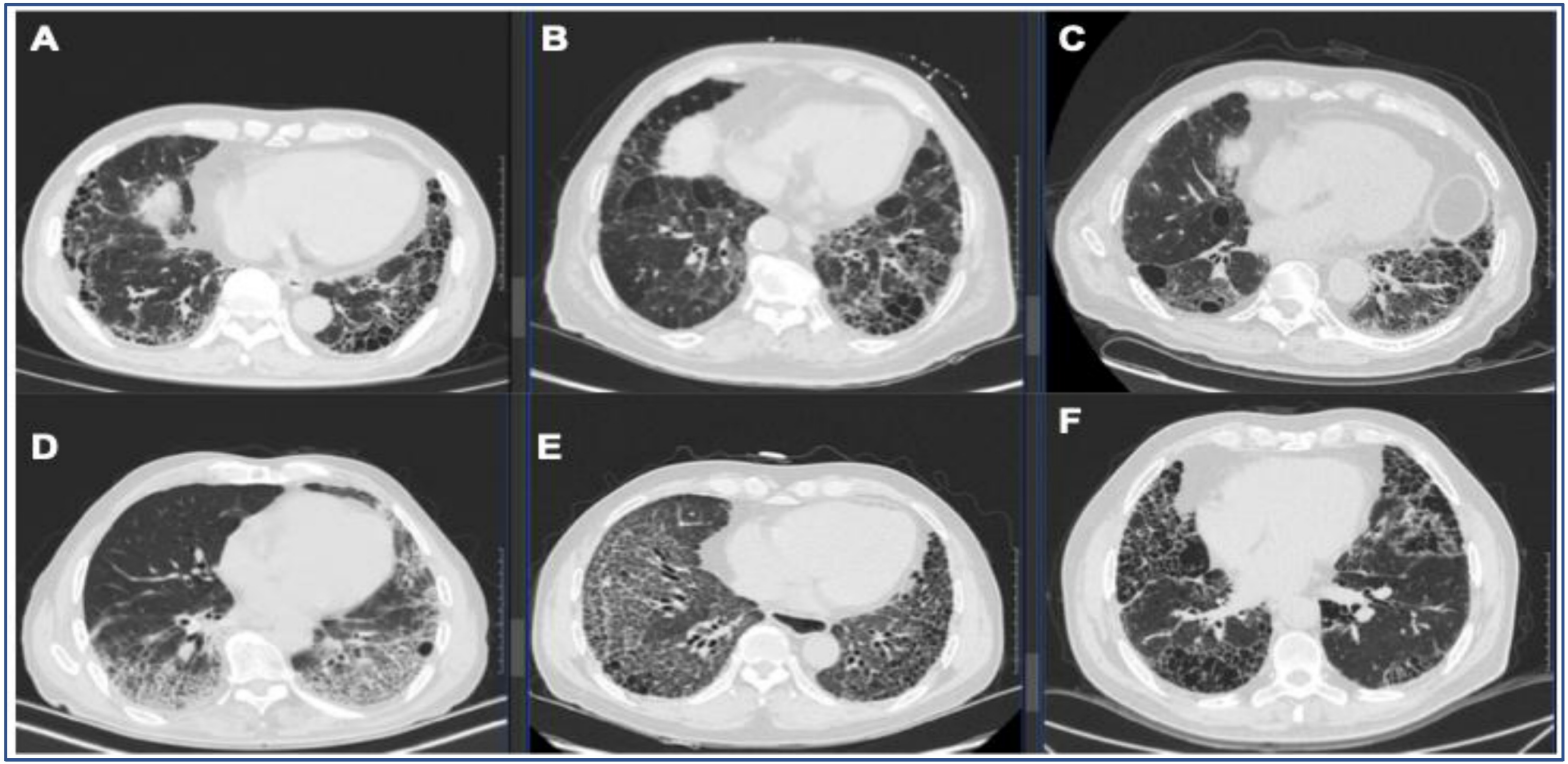
** Representative UIP pattern on HRCT in IPF and secondary UIP.** (A) IPF, (B) fHP, (C) RA-ILD, (D) pSS-ILD, (E) SSc-ILD, (F) AAV-ILD. Radiologic UIP pattern is defined as basilar, subpleural distribution of reticulation and traction bronchiectasis with honeycombing and without features incompatible with UIP.

The usual interstitial pneumonia (UIP) pattern is one of the most common radiological and histopathological features observed in fibrotic lung diseases, characterized by heterogeneity in the lung parenchyma, subpleural predominant reticulation and honeycombing 1. For decades, the UIP has been considered the pathognomonic hallmark of idiopathic pulmonary fibrosis (IPF), while other fibrotic lung diseases, including connective tissue disease-associated interstitial lung diseases (CTD-ILDs), interstitial pneumonia with autoimmune features (IPAF), fibrotic hypersensitivity pneumonitis (fHP), asbestosis, drug reactions, and familial pulmonary fibrosis, can also present UIP patterns (Figure 1, Figure 2). Discriminating idiopathic UIP (IPF) with secondary UIP is clinically significant for its potential therapeutic and prognostic implications. Management for fibrotic lung diseases differs significantly by etiology. While corticosteroids, immunosuppressants, and certain biological agents (e.g., rituximab) have shown efficacy in improving lung functions and slowing disease progression in patients with CTD-ILDs and fHP 2, antifibrotic treatment strategy remains the established standard of care for IPF patients for slowing the decline of FVC. The prognosis of fibrotic lung diseases also differs by etiology. Previous studies have demonstrated that with biopsy- or radiology- proven UIP, patients with IPF had worse survival (median survival 3.5-4.7 years) compared with fHP group (median survival 4.8 years), CTD-ILDs group (median survival 5.5-7.1 years) 3-6. Therefore, the differential diagnosis for the UIP pattern becomes particularly important in clinical care.

The underlying causes of the UIP pattern primarily encompass five clinical entities. IPF accounts for the largest proportion (54.15%-85.15%) 3-5, 7-9, followed by CTD-ILDs (10.46%-38.75%) 3, 5, 7-9, fHP (2.37%-6.60%) 5, 7, 8, asbestosis (about 4.74%) 5, and other (about 6.67%) 7, of which CTD can be subdivided into rheumatoid arthritis (RA) (22.71%-41.27%) 3-5, 9, primary sjögren's syndrome (pSS) (11.11%-31.00%) 4, 5, 9, anti-neutrophil cytoplasmic antibody associated vasculitis (AAV) (14.29%-25.33%) 4, 5, 9, systemic lupus erythematosus (SLE) (19.05%-19.64%) 4, and other CTD (14.28%-57.14%) including mixed CTD(mCTD), unclassifiable CTD (uCTD), and polymyalgia rheumatica etc 3-5, 9. Such broad ranges in proportional of underlying causes reflect significant heterogeneity across studies, primarily driven by population variance (e.g., geographic and environmental exposure differences), evolving diagnostic criteria and inherent referral bias or selection bias among different study cohorts (e.g., specialized interstitial lung disease centers versus general clinics).

Although distinctive CT signs have been proposed to differentiate UIP pattern of IPF from CTD-ILDs, i.e. anterior upper lobe sign, exuberant honeycombing, and straight edge sign 10, precisely diagnosing the underlying causes of UIP still remains challenging, especially at primary hospitals, albeit with growing understanding of IPF and secondary UIP. The effective diagnostic standard for IPF and secondary UIP is lacking, while current guidelines suggest multidisciplinary discussion (MDD) as a surrogate standard and ultimate choice 1, which can be unavailable at inexperienced centers.

Biomarker is measured as an indicator of normal biological processes, pathogenic processes or responses to an exposure or intervention. Biomarkers may exhibit promising applications in disease differentiation, monitoring and prognosis. This review focuses on the radiological UIP pattern and we aim to provide an evidence-informed overview of the risk factors and diagnostic biomarkers for UIP subtypes. Finally, we tend to discuss future research directions in artificial intelligence (AI) integrating with multimodal learning to realize precise diagnosis.

## UIP Pattern: Overlap and Distinction

### IPF

IPF is one of the most common fibrosing ILDs characterized by fatal lung function loss and progressive tissue remodeling. Radiological features of UIP or probable UIP, as defined by the guideline, is the hallmark of IPF 11. Diagnosis of IPF requires the exclusion of all other risk factors or exposures that are known to cause secondary UIP 1. The prevalence of IPF ranges from 14-27.9 cases to 42.7-63 cases per 100,000 people, depending on statistical methods and selection criteria 12. Risk factors include age, male sex, smoking, wood/ metal dust, sand/ silica, agricultural exposures and gastroesophageal reflux 13-15. Genomic risk factors include MUC5B promoter polymorphism rs35705950 16, TOLLIP 17, short telomere length 18, TLR3, IL1RN, IL-8, IL-4, TGFB1 19, DSP rs2076295 16 and AKAP13 16. FUT3, ADAM15 and USP28 are found to be possible genetic-informed proteomic risk factors 20. Recent evidence suggests that specific autoantibodies offer a distinct perspective for understanding IPF through immune mechanisms. Anti-periplakin autoantibodies target epithelial desmosomes and impair wound repair, while anti-HSPA2 antibodies may play a protective role 19, 21, 22. Diagnostic delay is widely observed in IPF, with median time being 2.1 years 23.

### fHP

HP is an immune-mediated, inflammatory parenchymal lung disease after inhalational exposure to an inciting antigen or mixture of antigens 24-26. It is estimated that the incidence of HP is 0.3-0.9 cases per 100,000 population, and the annual prevalence of HP is 1.67-2.71 cases per 100,000 individuals in the US 12. Current guideline propose a phenotype-based approach in definition, i.e. non-fHP and fHP 25. Definite or probable UIP pattern are observed in fHP patients, taking up 17% of fHP patients, and UIP pattern, whether histologically or radiologically proven, are often linked to worse survival similar to IPF 27, 28. MUC5B promoter polymorphism rs35705950, as a genomic risk factor in IPF patients, is also associated with HP incidence, UIP histopathology, and radiological fibrosis, explaining why fHP and IPF share certain common risk factors and clinical manifestations 27.

### RA-ILD

RA affects nearly 1% of the population 29, whereas RA-ILD is highly prevalent in RA cohorts (up to 60% 30). UIP or probable UIP pattern on CT are correlated with unfavorable outcomes similar to IPF, compared with other RA-ILD patients with alternative CT patterns 31-33 .Anti-cyclic citrullinated peptide (anti-CCP) positivity and titre are found to be strongly associated with RA-ILD susceptibility 34, while male sex, age at onset, cigarette smoking and rheumatoid factor titre are all risk factors independently associated with RA-ILD 35, 36. Higher levels of c-reactive protein, erythrocyte sedimentation rate (ESR), and tumor markers especially CA125 levels are also risk factors 37, 38. A recent study indicates that MUC5B promoter variant rs35705950 is associated with increased risk (at least 3-fold increased) of pulmonary involvement 39. A risk score system was developed and upgraded with MUC5B rs35705950 to improve model precision for subclinical RA-ILD 40. Genetic susceptibility to RA-ILD is significantly linked to variants in TOLLIP and HLA-DRB1 19. Several genomic characteristics observed in RA-ILD patients such as familial pulmonary fibrosis (FPF) linked genes (*TERT*, *RTEL1*, *PARN* or *SFTPC*) mutations and short telomeres that were previously linked with FPF susceptibility 41.

### SSc-ILD

SSc is characterized by vasculopathy and fibrosis in skin and multi-internal organs with a prevalence of 1 in 100,000 individuals, whereas ILD is observed in 25%-30% of SSc patients and acts as a major cause of mortality 42, 43. Older age, male sex, diffuse cutaneous SSc and cardio involvement are well-established risk factors for SSc-ILD 43, 44, while there is an uncertain association between cigarette smoking and susceptibility of SSc-ILD. UIP pattern accounts for less than 10% of patients in a retrospective cohort study but is linked with relatively faster lung function deterioration 45. Specific genetic variants, including STAT4 and IRF5, have been identified as key risk factors predisposing patients to SSc-ILD 19. MUC5B promoter polymorphism rs35705950 was not associated with SSc-ILD, suggesting its different pathophysiology with IPF 46.

### pSS-ILD

pSS is a common systemic autoimmune disease with a prevalence of 0.3-1 per 1000 individuals and a female predominance 47. Pulmonary involvement is a frequent manifestation of pSS and ILD is observed in 17% of pSS patients in a recent meta-analysis 48. Male sex remains one of the important risk factors for pSS-ILD 49, 50. Other identified risk factors include older age at onset and longer disease duration 48. Anti-SSA/Ro and anti-SSB/La antibodies are critical for diagnosing pSS-ILD for their specificity in pSS patients 51. However, current understanding on pSS-ILD is still limited. A particular diagnostic challenge arises when ILD precedes the formal diagnosis of pSS in individuals with subclinical disease, especially for those with a definite or probable UIP pattern on HRCT 52. This condition is challenging for clinicians and often results in misdiagnosis as unclassifiable ILD or IPF, with the correct diagnosis rate being only 4% 53. From a clinical standpoint, female sex, younger age, systemic manifestations and serological abnormalities are important discriminators between pSS-ILD and IPF.

### Microscopic polyangiitis (MPA)-ILD

MPA, a major subtype of AAV, is a pauci-immune necrotizing vasculitis with a prevalence of 3-10 cases per 1,000,000 individuals in the USA and Europe 54. ILD is observed in 2.7% to 47.4% of patients with MPA and often leads to worse survival 55. Age, male sex and smoking history are risk factors for MPA-ILD, and interestingly, *TERT* rs2736100A and *DSP* rs2076295G, both genomic risk factors for IPF, are associated with MPA-ILD, indicating that IPF and MAP may share some common pathogenic mechanisms 56, 57. UIP is the most frequent radiologic pattern in MAP-ILD patients, and is observed in more than 50% of MPA-ILD patients 55, 56, 58, 59. Serological test for MPO-ANCA is critical for diagnosis, which is found in over 80% MPA-ILD patients and shows high specificity in differential diagnosis 60, 61. However, pulmonary manifestations can precede the onset of systemic vasculitis symptoms, resulting in an initial misdiagnosis as IPF and delaying the treatment for MPA 56. The clinical features of idiopathic UIP and common secondary UIP diseases are summarized in Table 1.

### Diagnostic biomarkers

#### IPF

Genetic variants influence IPF susceptibility, progression and prognosis. The MUC5B polymorphism rs35705950 is known to be the strongest genetic risk factor with a 14-fold increased risk for IPF. However, its prognostic value remains a subject of debate, with conflicting evidence observed across different studies. Previous studies have reported that carriers of the MUC5B risk allele exhibit slower disease progression and improved survival compared to non-carriers 27, 62. Conversely, recent studies demonstrate conflicting results, indicating that this genetic variant may not always consistently correlate with better survival outcomes 63-65. These disparate findings suggest that the prognostic value of MUC5B remains a matter of controversy influenced by many reasons. In contrast, TOLLIP variant (rs5743890 minor allele) accelerates the progression of lung fibrosis and leads to worse survival 17. Telomere length is associated with fibrosis extent, and shortened telomeres (lengths less than the 10th percentile for age) result in cellular senescence, rapid disease progression and reduced survival 27.

Serological biomarkers for IPF diagnosis can be categorized into a few groups based on their association with core pathological mechanisms: alveolar epithelial injury (e.g., SP-D, CA19-9, CA-125), extracellular matrix remodeling (e.g., MMP-1, MMP-7, Osteopontin), and immune/chemokine signaling and fibrotic mediators (e.g., CCL17, BPI, LTBP2). What's more, protein signatures (e.g., PC37, a 5-protein panel) and microRNA profiles demonstrate diagnostic utility in discriminating IPF from healthy controls and other ILDs. Biomarkers associated with IPF are summarized in Table 2.

Novel biomarkers such as electronic Nose (eNose) and genome UIP (gUIP) have shown adequate specificity and sensitivity in differentiating ILD subtypes. Exhaled breath analysis-based eNose technology is a promising non-invasive biomarker to discriminate healthy individuals from various ILD subgroups by analyzing different chemical compounds in volatile organic compounds (VOCs) in exhaled breath 66. Derived from transbronchial biopsies, gUIP has been validated in predicting histopathological UIP and increasing diagnostic confidence for IPF, and positive gUIP classification is associated with reduced transplant free survival (TFS) 67. Biomarkers for diagnosis of IPF (Table 2).

#### fHP

A large-scale study of single-cell immune profiles revealed novel immune perturbations in fHP. Both fHP and IPF share enriched S100Aʰⁱ and CCL3ʰⁱ/CCL4ʰⁱ classical monocytes whereas cytotoxic GZM^hi^ lymphocytes are enriched specifically in fHP and may contribute to disease discrimination. The study also found variance in cell compositions in both blood and BAL fluid between fHP and IPF patients 68. Compared to IPF, fHP shows increased classical monocytes and platelets but decreased memory B cells, Tregs, MAIT cells, and CD56ʰⁱ NK cells in blood and fewer alveolar macrophages but more T cells in BAL fluid 68. Previous studies have verified SP-D as an alveolar epithelial injury marker for HP diagnosis. New evidence identifies YKL-40 and KL-6 as viable candidates with adequate specificity and sensitivity to discriminate HP from healthy controls and IPF patients. Differences in microbiome patterns in fHP and IPF also contribute to discrimination 69. Biomarkers associated with fHP are summarized in Table 3.

### CTD-ILDs

The biomarker landscape for CTD-ILDs is rapidly evolving, encompassing markers of genetic risk (MUC5B 39), autoimmunity (ACPA, Citrullinated HSP90), inflammation (chemokines), cellular senescence (telomere length), epithelial injury (KL-6, SP), and matrix remodeling (MMP-7, LOXL2). A recent study suggests Reticulocalbin 3 (Rcn3), an endoplasmic reticulum lumen protein concerning alveolar epithelial injury, holds promise in discriminating CTD-ILDs from IPF, for serum Rcn3 concentration was significantly higher in patients with CTD-ILDs than in those with IPF; yet, further studies are still needed 70.

A few biomarkers contribute to pSS-ILD diagnosis. A machine learning based study identified age, disease duration, and serum levels of KL-6 and TNFα as highly discriminating biomarkers for pSS-ILD and pSS-non-ILD 71. A recent study investigated compositional and functional similarities and differences of the gut microbiota in pSS and SLE patients, suggesting the potential role of microbiome profiling in CTD-ILDs diagnosis. 72

Current research on biomarkers for MPA-ILD remains limited. MPO-ANCA, while being a cornerstone serological marker for MPA and demonstrating high specificity in distinguishing MPA-ILD from other ILDs, is shared across AAV (GPA, MPA, EGPA included) and does not specifically reflect pulmonary involvement or disease progression 61, 73. Significantly elevated serum CCL2 levels in MPA-ILD patients enable discrimination between MPA-ILD patients, MPA-non-ILD patients and healthy individuals 74. Biomarkers correlating to alveolar epithelial injury, including KL-6 and SP, reflect lung-specific activity and are useful in monitoring pulmonary lesions, but their utility in diagnosing and monitoring MPA-ILD are limited 75. Biomarkers associated with CTD-ILDs are summarized in Table 4.

### Artificial intelligence (AI)-driven biomarker discovery for diagnosis

The application of AI and, more specifically, machine learning (ML) has revolutionized the discovery of biomarkers for ILDs. By integrating multi-omics data and dealing with large-scale biomedical data (such as genomic sequencing and proteomics) simultaneously, AI-assisted analytical tools enable a systems-level, data-driven paradigm that integrates multimodal information 76.

ML-based techniques have been applied in plasma and serum proteomics to identify novel circulating biomarkers in ILDs, where they now outperform the classic and established biomarkers. AI-driven ILD biomarkers have been proven efficient in some real-world case studies. Bowman et al. applied LASSO (least absolute shrinkage and selection operator) logistic regression and ten-fold cross-validation to identify a set of biomarkers predictive of progressive fibrosing ILDs. A semi-quantitative proteomic signature comprising twelve biomarkers achieved a sensitivity of 0.90 in discriminating progressive and non-progressive ILD patients 77. Huang et al. performed SVM, LASSO and RF to develop and validate a proteomics classifier with 37 proteins (PC37) associated with bronchiole development and immune responses, demonstrating 82.9% accuracy in discriminating CTD-ILDs and IPF. By iteratively classifying single samples and applying composite scoring methods across all four machine learning models, they established a single-patient diagnosis model mimicking clinical practice settings 78. Zhou et al. developed a pulmonary fibrosis model using RF and SVM. Three key genes including TMEM52B, PHACTR1 and BLVRB were selected using RF and SVM found that the model achieved an accuracy of 0.786 when 15 genes were incorporated, providing a new solution for early diagnosis of pulmonary fibrosis 79. Oldham et al. identified 140 circulating plasma proteins associated with differential transplant-free survival (TFS) in IPF cohorts. LASSO was applied to build a proteomic signature of TFS and the model performance was estimated using decision curve analysis. The model significantly outperformed a clinical prediction model on discriminating TFS, showing its potential in clinical decision-making and disease monitoring 80.

The identification of novel biomarkers with AI assistance involves a multi-step, rigorous process. Well-designed clinical cohorts have to be recruited and participants have to be categorized or phenotyped, discovery cohort and validation cohort have to be randomly assigned after sample preprocessing. Either mass spectrometry (MS) or high-throughput proteomics platforms can be applied in data acquisition. Omic data and/ or clinical data are appropriately collected and stored integrated and pre-processed. The processed data are divided into a training and a testing dataset. Having obtained such extensive data sets, the next step is univariate and/ or multivariate statistical analyses to quantify the proteome differences between cases and controls, often visualized through volcano plots and heatmaps. We hereby select candidate differentially accumulated metabolites (DAMs) and conduct cross validation. Then we train the ML models (LASSO, RF, SVM-RFE, LR, XGBoost, etc) to obtain optimal parameters and tune hyperparameters. The learning approach (supervised, semi-supervised, unsupervised) is steered by the ultimate goals and the availability of collected data. Evaluation of biomarkers found by ML and ML models should be done in the following steps, such as with confusion matrices and ROC curves, which quantify the sensitivity and specificity of candidate biomarkers and facilitate their potential translation into clinical settings. The predictive power and explainability of the biomarker are validated on external datasets to assess its robustness and generalizability on unseen data (usually from another medical center).

To ensure the robustness and reproducibility of the AI-driven workflow, we recommend a few technical details as indicated by Mann et al., which adheres to the best practices for machine learning in proteomics 76. Good ML models should be configured with explicit hyperparameter settings, such as learning rate or specific coefficients to control model complexity. We suggest a strict splitting of training and evaluation data, where the evaluation data are never employed in training. Usually a training set (~70%), an internal validation set (~15%), and an independent external testing cohort (~15%) is ideal. Of note, the accurate quantification of the biomarkers is even more critical than the identification of as many as possible of them, and when samples are really restricting, overfitting prevention methods such as cross-validation are essential for robust evaluation of model performance. K-fold cross-validation and regularization techniques (i.e., Ridge regression) are also procedures for preventing overfitting and confusion matrices and ROC curves are often applied for biomarker evaluation 76.

The AI models and biomarker discovery also have limitations. First, the explainability of DL and ML models is crucial, as ML and DL models often contain thousands of nodes and often lose interpretability; hence, ML and DL models are often treated as black boxes with no clinical significance. Beyond that, it would also be troublesome when considering discordance between the AI classifier and MDD 78, thus it is important to address these challenges through explainable AI (XAI) and more in-depth model evaluation to determine how well the AI classifier correlates with real-world patient diagnosis.

## Concluding remarks and future directions

UIP is a well-established terminology back to 1969. The emphasis and usage of the term UIP has shifted over decades, but with obvious limitations. The designation of UIP remains ambiguous in a series of conditions 81. Changes in guidelines (from 2011 to 2018 and 2022 updates) aim to reduce heterogeneity but still result in inconsistent classification. The classification of UIP into “typical,” “probable,” “indeterminate,” or “alternative diagnosis” relies heavily on personal experience and expertise. In a study concerning a group of thoracic radiologists, significant interobserver variance and disagreement exist, with only moderate consensus achieved, irrespective of their expertise or experience 82. Current guidelines clarify UIP as a pathologic fibrotic pattern, not synonymous with IPF, but terms like “UIP pattern” or “IPF” are often used interchangeably, causing confusion and misleading outcomes. Shifting diagnostic focus and developing new classifications are still needed.

This review summarizes underlying causes, risk factors, clinical features and diagnostic biomarkers of idiopathic UIP and secondary UIP subtypes. UIP pattern can be found in a series of conditions, but recently, it has been proposed that UIP be defined as a distinct disease entity, due to similarities in disease behaviors and pathogenic mechanisms 83. However, we have to recognize that the clinical trials are based on large patient cohorts, not individuals. Therefore, when dealing with patients with histological or radiological UIP pattern, the primary task is to separate idiopathic UIP (IPF) from secondary UIP, mainly fHP and CTD-ILDs.

The biomarkers we described here are obvious candidate biomarkers based on our current understanding of diseases. Biomarkers are less invasive and more objective indicators, crucial for early diagnosis, monitoring treatment response, and prognostication. Exploring biomarkers may just be the key we need in unlocking the complex nature of UIP, allowing for early-stage interventions that could change patients' lives.

Despite the fact that considerable advances have been made and substantial biomarkers have been observed and advanced techniques such as single-cell sequencing and multi-omics data integration offer unprecedented insights, their application into routine clinical practice is currently hindered by infrastructure, technical standardization and reproducibility, population heterogeneity and generalizability, high costs and translational challenges, especially in primary clinical settings. To bridge this gap, a biomarker must be measurable, accurate, reproducible, clinically actionable, and cost-effective. Beyond that, the selection and combination of biomarkers should be guided by a clear framework: prioritizing widely available serum markers for longitudinal monitoring, while reserving high-specificity proteomic or metabonomic signatures for complex differential diagnosis. Balancing feasibility, diagnostic validity, and the specific clinical context will be essential for improving patient quality of life and survival substantially.

These studies on biomarkers also have limitations and deficiencies. The variance on disease definition and clinical diagnosis criteria is the primary reason. What's more, statistical manipulation and overfitting also occur. Inappropriate statistical methods can cause bias and misleading results. Although many studies used a multivariate Cox proportional hazards model to adjust for potential confounders, others relied on univariate analyses. The application of univariate methods is a significant limitation, for their defects in capturing the data's complex, multi-dimensional patterns. Thus, it is essential for biomarker studies to employ multivariate analysis (e.g., Cox proportional hazards models or logistic regression) to adjust for key clinical variables including age, sex, BMI, smoking history, medication use and comorbidities to ensure that the identified biomarkers are independent predictors of disease progression and outcomes rather than reflections of underlying clinical characteristics. Insufficient sample sizes and retrospective bias also contribute to it. Future studies should improve data standardization, reproducibility, cross-platform validation, and prioritize AI integration in multi-omics data to provide new insights in novel biomarkers.

## Figures and Tables

**Figure 3:**
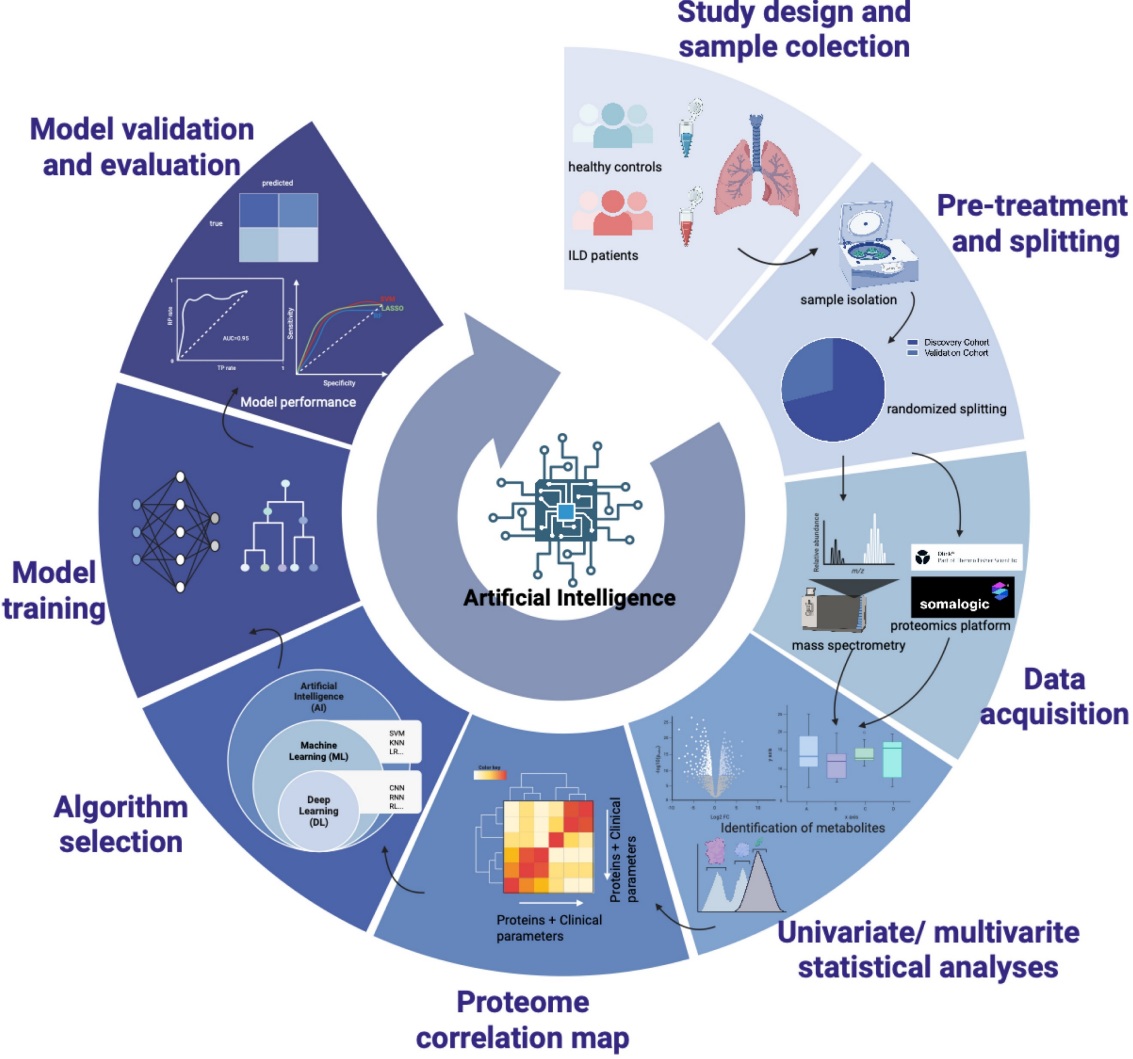
** AI-driven workflow for proteomic biomarker discovery and model validation in ILDs.** This integrated pipeline encompasses seven key stages of development. First, study design and sample collection involve cohorts of healthy controls and ILDs patients. Second, pre-treatment and splitting are performed to divide samples into discovery and validation cohorts. Third, data acquisition utilizes high-throughput technologies such as mass spectrometry and the Somalogic proteomics platform. Fourth, univariate and multivariate statistical analyses are applied for metabolite identification and data processing. Fifth, a proteome correlation map is generated to integrate protein data with clinical parameters. Sixth, algorithm selection involves choosing between machine learning and deep learning. Finally, model training is followed by rigorous model validation and evaluation using performance metrics such as AUC and sensitivity-specificity curves. Created in BioRender.com, with permission.

**Table 1:** Clinic features of Idiopathic UIP (IPF) and Secondary UIP.

	Idiopathic UIP	Secondary UIP				
	IPF	fHP	CTD-UIP			
			RA-ILD	SSc-ILD	pSS-ILD	MPA-ILD
**Age**	Most patients diagnosed with IPF are over 60 years old 84	The average age of onset is 52 years, and higher risk for individuals over 65 85	Most diagnosed at sixth decade of life, and increased mortality risk after 64.7	Median age of 60.7 years old 86	Peak incidence at 55-61 47, 53	Most diagnosed over 65 years 58
**Sex**	Males represent ∼70% of all patients with IPF 6, 87	Males represent fewer than half of the cases 85	Males are affected twice as frequently as females 88	Female predominance in absolute numbers (over 75% female patients) 86	Female predominance in absolute numbers (about 90% female patients) 47	Higher frequency of men 56
**Cigarette smoking**	Ever-smokers account for more than 50% of IPF patients 14, 89	Less frequent in ever-smokers than non-smokers, and smoking is associated with lower survival rates. 90	Smoking over 25 pack years have 2.4 times greater chances of demonstrating lung erosions 34	Unknown association	Ever-smokers account for about 20% of pSS-ILD patients 53	Higher frequency of smokers, and smoking is associated with higher mortality 56
**UIP pattern**	Hallmark of diagnosis 1	Observed in 17% of fHP patients 27	Over 50% of RA-ILD patients 91, 92	Less than 10% of patients 45	About 10% of patients 53	More than 50% of patients 55, 56, 58, 59
**Gastro-esophageal reflux**	Associated risk factor 13	Unknown association	Unknown association	Present in 80% SSc-ILD patients 93	Present in 50% pSS-ILD patients 53	Unknown association
**Exposures**	Associated with environmental factors such as agriculture, farming, livestock, wood dust, and metal dust 94	Antigen exposures are essential components in diagnosis, including microbes, proteins, enzymes, chemicals and medication 25	Associated with inhalational exposures, include silica, stone dust, rock drilling or stone crushing 95	Unknown association	Unknown association	Unknown association
**Obstructive sleep apnea**	Highly prevalent in IPF 96, 97	Unknown association	Unknown association	Highly prevalent 98	OSA observed in about 30% of pSS patients 99	Unknown association
**Acute exacerbation (AE)**	AE occurs, with 1-year, 2-year and 3- year incidence of 14.2%, 18.8% and 20.7% respectively 100	AE occurs, with 1-year and 3-year incidence of 6.0%, 13.6% and 22.8% respectively 101	AE occurs, with 1-year incidence of 2.8% 102	AE occurs, but the incidence not fully studied	AE occurs, but the incidence not fully studied 103	AE occurs, with 1-year incidence of 7.5% 56
**Prognosis**	Median survival 3.5-4.7 years 4, 6	Median survival 2.76 years 28	Median survival 5.5 years 3	Median survival 13.5 years 104	5-year survival rate 84% 53	5-year survival rate 29%-60% 58
**Immune suppression based treatment strategy**	Proven to associated with shorter survival and high mortality 2	Immunosuppressants is associated with increased mortality 105. Corticosteroid has no influence on long-term results 106	Leflunomide is associated with better prognosis 32	Mycophenolate mofetil, cyclophosphamide, and tocilizumab 107	Azathioprine and prednisone 108	Glucocorticoid and cyclophosphamide 109
**Anti-fibrotic treatment strategy**	Slow lung function decline 110, 111	Evaluation of efficiency and safety is still in need.	Slow lung function decline 112	Slow lung function decline 113, 114	May be effective, but not proven 115	May be effective, but not proven 56

**Table 2:** Biomarkers for diagnosis of IPF.

Biomarkers	Clinical utility /Results	Reference
SP-D	SP-D > 31ng/mL differentiate IPF from other-ILD and healthy controls	116
CA19-9, CA-125	CA19-9 and CA-125 discriminate IPF and other ILDs	116
MMP-1, MMP-7	MMP-7 > 1.75ng/mL distinguish IPF from other-ILD, and MMP-7 > 12.1ng/mL are predictive mortality and TFS	117, 118
	MMP-1 ≥ 1.99ng/mL and MMP-7 ≥ 2.15ng/mL distinguish IPF from other ILDs	
Osteopontin	Osteopontin > 6ng/mL distinguish IPF and other-ILDs	117
GT1, vWF, CCL17, BPI	Circulating proteins (GT1, vWF, CCL17 and BPI) differentiate IPF and other healthy controls	119
LTBP2	Higher levels in IPF than seen in CTD-ILDs	117
	LTBP2 > 33.75 ng/mL discriminate IPF and CTD-UIP (AUC 0.682)	
	LTBP2 > 38.33 ng/mL discriminate IPF and RA-UIP (AUC 0.681)	
cCCK18	Serum cCK-18 levels are elevated in IPF patients and may be clinically informative as a diagnostic marker of IPF	120
LDHA, CCT6A	LDHA and CCT6A differentiate IPF from healthy controls	15
Protein signature	A combinatorial signature of five proteins (MMP7, MMP1, MMP8, IGFBP1, and TNFRSF1A) discriminates IPF from healthy controls with a sensitivity of 98.6% and specificity of 98.1%	121
PC37	A classifier with 37 proteins consistently distinguishes CTD-ILDs from IPF	78
eNose	eNose technology can be a novel biomarker for its potential to discriminate IPF and other ILDs	66
microRNAs	Downregulated micro RNAs (miR-29, let-7d) and upregulated micro RNAs (miR-21, miR-154) are shown to have diagnostic capacity in IPF	122

Abbreviations: LTBP2, Latent transforming growth factor-beta binding protein-2; SP-D, Surfactant protein D; MMP-7, Matrix metalloproteinase-7; PC37, Proteomic classifier 37; TFS, Transplant free survival; LDHA, Lactate dehydrogenase A; CCT6A, Chaperonin containing TCP1 subunit 6A; GT1, Glycoproteins thrombospondin 1; vWF, von Willebrand factor; CCL17/18, C-C motif chemokine ligand 17/18; BPI, Bactericidal permeability-increasing protein; IGFBP1, insulin-like growth factor binding protein1; TNFRSF1A, tumor necrosis factor receptor superfamily member, 1A.

**Table 3:** Biomarkers for diagnosis of fHP.

Biomarkers	Clinical utility /Results	Reference
S100^hi^ monocytes, CCL3^hi^/CCL4^hi^ monocytes	S100^hi^ monocytes and CCL3^hi^/CCL4^hi^ monocytes are both highly enriched in fHP patients with the potential to discriminate fHP and healthy individuals.	68
GZM^hi^ lymphocytes	Cytotoxic GZM^hi^ lymphocytes are increased in fHP compared with healthy controls and IPF patients with the potential for discrimination between fHP and others.	68
SP-D	Serum SP-D level in HP are higher than in IPF, CTD-ILDs and sarcoidosis and SP-D is useful for diagnosis.	123
YKL-40, KL-6	Serum and sputum YKL-40 and KL-6 levels in fHP are higher than healthy individuals while lower than IPF, with adequate specificity and sensitivity for diagnosis.	124
Microbiome	Differences in bacterial community composition and diversity contribute to discrimination of fHP and IPF.	69

Abbreviations: CCL3/4, C-C motif chemokine ligand3/4; GZM, Granzyme; SP-D, Surfactant protein D; YKL-40(CHI3L1), Chitinase 3-like protein 1; KL-6, Krebs von den lungen-6

**Table 4:** Biomarkers for diagnosis of CTD-ILDs.

	Biomarkers	Clinical utility /Results	Reference
RA-ILD	Uric Acid	Elevated serum and BAL fluid uric acid levels may serve as a diagnostic biomarker for RA-ILD, particularly RA-UIP.	125
	Citrullinated Hsp 90α/β	Citrullinated Hsp 90α/β can differentiate RA-ILD from RA-non-ILD, IPF and MCTD.	126
	ACPA	ACPA differentiates RA-ILD from RA-non-ILD, and associates with worse survival.	36
	LOXL2	Serum LOXL2 levels are higher in RA-ILD patients than healthy individuals, with potential utility in diagnosis.	127
	MMP-3, MMP-7, MMP-9, MMP-10, TIMP-1	MMPs and TIMPs found as potential candidates for the discrimination of RA-ILD and IPF.	128
pSS-ILD	KL-6, TNF-α	Age, KL-6, and TNFα effectively differentiated pSS-ILD from pSS-non-ILD with high sensitivity and specificity.	71
	CA153	Serum CA153 levels are higher in pSS-ILD patients than pSS-non-ILD.	129
	Angptl2	Serum Angptl2 level are highly elevated in pSS-ILD patients compared with healthy controls and pSS-non-ILD patients.	130
	CYSLTR1, SIGLEC10	Differential expression of CYSLTR1 and SIGLEC10 in pSS-ILD and healthy controls revealed their diagnostic potential for pSS-ILD	131
MPA-ILD	CCL2	Initial serum CCL2 levels were significantly higher in MPA-ILD patients than MPA-non-ILD patients.	132
	KL-6	Higher serum KL-6 were in MPA-ILD compared with those without ILD, and serum KL-6 > 513 U/mL differentiates MPA-ILD with other AAV.	133

Abbreviations: ACPA, Anticitrullinated protein antibody; LOXL2, Lysyl oxidase-like 2; MMPs, Matrix metalloproteinases; TIMPs, Tissue inhibitor of metalloproteinases; KL-6, Krebs von den lungen-6; CCL2, C-C motif chemokine ligand 2; CA153, Cancer antigen 153; TNF-α, Tumor necrosis factor-alpha; Angptl2, Angiopoietin-like protein 2; CYSLTR1, Cysteinyl Leukotriene Receptor 1; SIGLEC10, Sialic acid binding Ig like lectin 10

## Abbreviations

UIP: Usual interstitial pneumonia
CTDs: Connective tissue diseases
IPF: Idiopathic pulmonary fibrosis
fHP: fibrotic hypersensitivity pneumonitis
CTD-UIP: Connective tissue disease associated UIP
IPAF-UIP: Interstitial pneumonia with autoimmune features associated UIP
RA: Rheumatoid arthritis
pSS: primary Sjogren's syndrome
IIM: Idiopathic inflammatory myopathy
SLE: Systemic lupus erythematosus
SSc: Systemic sclerosis
AAV: Anti-neutrophil cytoplasmic antibody associated vasculitis
MCTD: Mixed connective tissue disease
UCTD: Undifferentiated connective tissue disease
LTBP2: Latent transforming growth factor-beta binding protein-2
SP-D: Surfactant protein D
MMP-7: Matrix metalloproteinase-7
PC37: Proteomic classifier 37
TFS: Transplant free survival
LDHA: Lactate dehydrogenase A
CCT6A: Chaperonin containing TCP1 subunit 6A
GT1: Glycoproteins thrombospondin 1
vWF: von Willebrand factor
CCL17/18: C-C motif chemokine ligand 17/18
BPI: Bactericidal permeability-increasing protein
IGFBP1: insulin-like growth factor binding protein1
TNFRSF1A: tumor necrosis factor receptor superfamily member: 1A
CCL3/4: C-C motif chemokine ligand3/4
GZM: Granzyme
YKL-40(CHI3L1): Chitinase 3-like protein 1
KL-6: Krebs von den lungen-6
ACPA: Anticitrullinated protein antibody
LOXL2: Lysyl oxidase-like 2
MMPs: Matrix metalloproteinases
TIMPs: Tissue inhibitor of metalloproteinases
CCL2: C-C motif chemokine ligand 2
CA153: Cancer antigen 153
TNF-α: Tumor necrosis factor-alpha
Angptl2: Angiopoietin-like protein 2
CYSLTR1: Cysteinyl Leukotriene Receptor 1
SIGLEC10: Sialic acid binding Ig like lectin 10
